# Wet-Chemically Prepared Porphyrin Layers on Rutile TiO_2_(110)

**DOI:** 10.3390/molecules26102871

**Published:** 2021-05-12

**Authors:** Daniel Wechsler, Cynthia Carolina Fernández, Julia Köbl, Lisa-Marie Augustin, Corinna Stumm, Norbert Jux, Hans-Peter Steinrück, Federico José Williams, Ole Lytken

**Affiliations:** 1 Lehrstuhl für Physikalische Chemie II, Friedrich-Alexander-Universität Erlangen-Nürnberg, Egerlandstraße 3, 91058 Erlangen, Germany; daniel.wechsler@fau.de (D.W.); julia.koebl@fau.de (J.K.); lisa-marie.augustin@fau.de (L.-M.A.); hans-peter.steinrueck@fau.de (H.-P.S.); 2 Departamento de Química Inorgánica, Analítica y Química Física, Facultad de Ciencias Exactas y Naturales, INQUIMAE-CONICET, Universidad de Buenos Aires, Ciudad Universitaria, Pabellón 2, Buenos Aires C1428EHA, Argentina; cfernandez@qi.fcen.uba.ar (C.C.F.); fwilliams@qi.fcen.uba.ar (F.J.W.); 3 Lehrstuhl für Katalytische Grenzflächenforschung, Friedrich-Alexander-Universität Erlangen-Nürnberg, Egerlandstraße 3, 91058 Erlangen, Germany; corinna.stumm@fau.de; 4 Lehrstuhl für Organische Chemie II, Friedrich-Alexander-Universität Erlangen-Nürnberg, Nikolaus-Fiebiger-Str. 10, 91058 Erlangen, Germany; norbert.jux@fau.de

**Keywords:** porphyrins, oxide surfaces, wet chemical preparation, metalation, interfaces

## Abstract

Porphyrins are large organic molecules that are interesting for different applications, such as photovoltaic cells, gas sensors, or in catalysis. For many of these applications, the interactions between adsorbed molecules and surfaces play a crucial role. Studies of porphyrins on surfaces typically fall into one of two groups: (1) evaporation onto well-defined single-crystal surfaces under well-controlled ultrahigh vacuum conditions or (2) more application-oriented wet chemical deposition onto less well-defined high surface area surfaces under ambient conditions. In this study, we will investigate the wet chemical deposition of 5-(monocarboxyphenyl)-10,15,20-triphenylporphyrin (MCTPP) on well-defined rutile TiO_2_(110) single crystals under ambient conditions. Prior to deposition, the TiO_2_(110) crystals were also cleaned wet-chemically under ambient conditions, meaning none of the preparation steps were done in ultrahigh vacuum. However, after each preparation step, the surfaces were characterized in ultrahigh vacuum with X-ray photoelectron spectroscopy (XPS) and the result was compared with porphyrin layers prepared in ultrahigh vacuum (UHV) by evaporation. The differences of both preparations when exposed to zinc ion solutions will also be discussed.

## 1. Introduction

**Figure 1:**
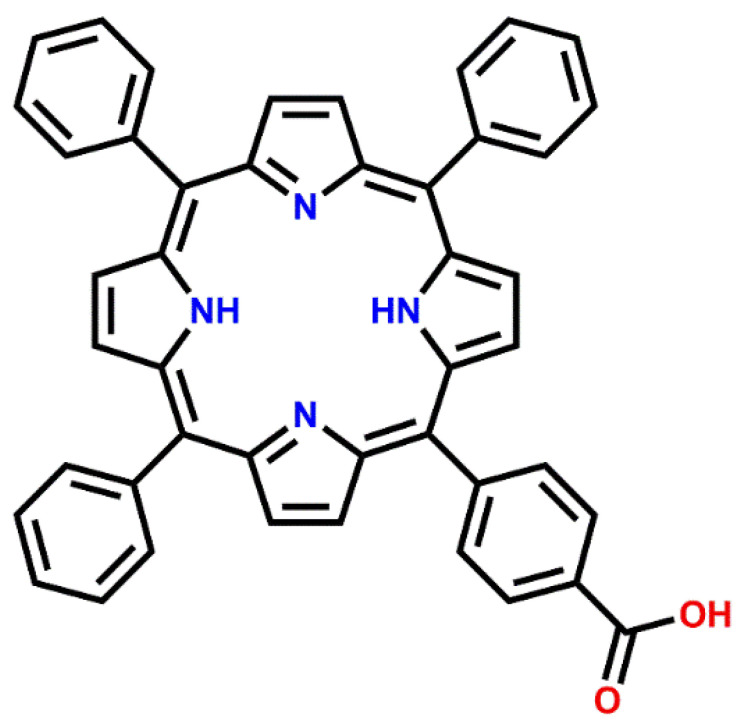
Chemical structure of 5-(monocarboxyphenyl)-10,15,20-triphenylporphyrin (MCTPP).

Porphyrins are large organic macrocycles, which play a crucial role in many important processes in nature [1,2]. These colorful molecules, sometimes referred to as the “pigments of life” [3], are the functional building blocks in hemoglobin and myoglobin, where they transport and store oxygen in mammalian cells [4], in chlorophyll, where they absorb sun light [5], and in vitamin B_12_, where they play an important role for the production of red blood cells and the function of the nervous system [6]. The reason for their broad range of functionality is their great tunability: by incorporating different metal centers in the nitrogen pocket, and by changing the side groups of the molecule, it is possible to tailor porphyrin derivatives with specific electronic, optical, and chemical properties [7,8,9]. This makes this group of molecules also highly relevant for catalysis [10,11], medical applications [12,13], and devices, such as gas sensors [14,15] and photovoltaic cells [16,17]. In many of these applications, porphyrins are adsorbed onto a solid support and, therefore, it is crucial to understand the interactions between porphyrin molecules and surfaces. The vast majority of existing surface science studies have focused on porphyrins adsorbed on metal surfaces [18,19,20,21,22], but in the last years, focus has shifted towards the more complex and more technologically relevant porphyrin/metal oxide interfaces [23,24,25]. These studies fall usually in one of two categories: (1) evaporation onto well-defined single crystal surfaces under well-controlled ultrahigh vacuum (UHV) conditions [23,24,25] or (2) more application-oriented wet chemical deposition onto less well-defined high surface area surfaces under ambient conditions [26,27]. In this study, we want to bridge the gap between these two categories: We focus on reproducibly growing porphyrin layers from solution onto wet-chemically cleaned TiO_2_(110) and the reaction of those layers with metal ion-containing solutions, all done under ambient conditions outside the vacuum chamber. Each step of the process will be characterized using X-ray photoelectron spectroscopy (XPS). We will furthermore compare the wet-chemically prepared porphyrin layers on TiO_2_(110) with layers grown under ultrahigh vacuum by evaporation onto TiO_2_(110) crystals, which were previously cleaned by sputtering and annealing. The porphyrin molecule we used for these measurements is the carboxylic acid-functionalized 5-(monocarboxyphenyl)-10,15,20-triphenylporphyrin (MCTPP, see Figure 1), which can be evaporated in ultrahigh vacuum [24,28] and is soluble in ethanol.

## 2. Results and Discussion

### 2.1. Preparation of the TiO_2_(110) Crystals

**Figure 2:**
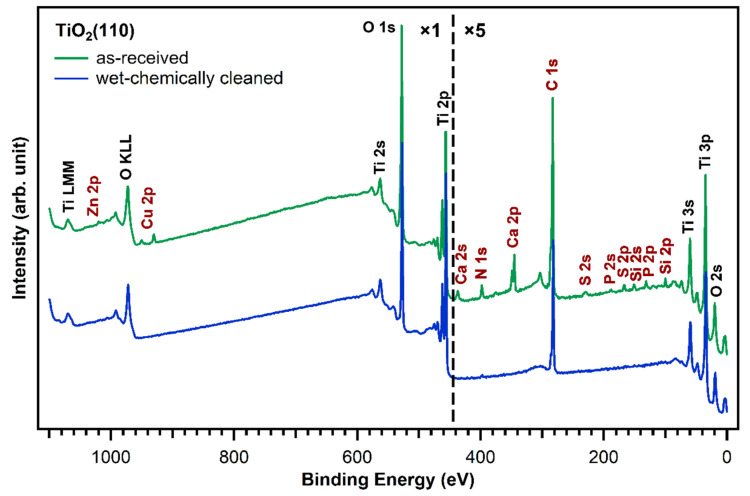
Comparison of XP survey spectra of a fresh, as-received TiO_2_(110) crystal (green) and a cleaned one (blue). Besides titanium and oxygen, traces of zinc, copper, calcium, sulfur, phosphorus, silicon, carbon, and nitrogen were found on the as-received crystal and removed by the wet-chemically cleaning procedure.

Figure 2 shows XP survey spectra of the as-received TiO_2_(110) crystal (green curve) and after subjecting the crystal to the wet chemical cleaning procedure described in the experimental section above (blue curve). The measurements of the untreated, as-received TiO_2_(110) crystal show—besides titanium and oxygen—the presence of zinc, copper, calcium, sulfur, phosphorus, silicon, nitrogen, and carbon contaminant species on the crystal surface. The elemental compositions of the as-received and wet-chemically cleaned crystals are shown in Table 1. All concentrations are given as atomic % within the XPS probing depth. Although all of these contaminants will affect the deposition of a porphyrin layer to different degrees, zinc and copper are particularly unwanted as they can be complexated by porphyrins and only small amounts are sufficient to metalate a full first layer of free-base porphyrins [29,30]. Indeed, the metalation of 1 ML of porphyrins (4 atomic % nitrogen, see Table 1) requires only 1 atomic % of reactive metals on the surface, as every porphyrin molecule consists of four nitrogen atoms, which can coordinate one metal atom. The as-received TiO_2_(110) crystal has already 0.82 atomic % copper and 0.12 atomic % zinc. Together, this is enough to metalate 0.9 ML of porphyrin molecules. Thus, it is very important to remove all metal impurities before free-base porphyrins are deposited. Moreover, the presence of nitrogen impurities is unwanted as they could give rise to XPS peaks that overlap with the porphyrin nitrogen peaks, making the interpretation of this spectral region very difficult.

**Figure 3:**
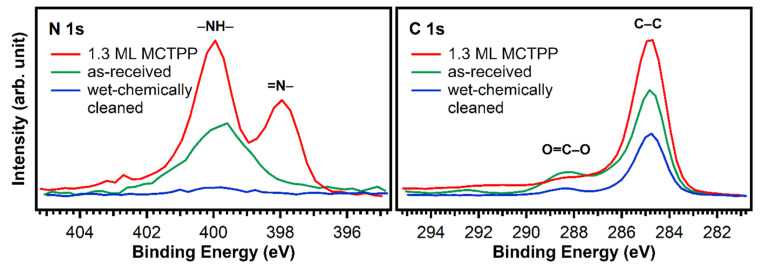
N 1s and C 1s spectra of a fresh and untreated TiO_2_(110) crystal (green) and a wet-chemically cleaned one (blue). In addition, the spectrum of a wet-chemically cleaned crystal exposed to MCTPP/EtOH-solution for 30 min is added (red) as a reference. The measurements show that the cleaning procedure removes almost all nitrogen, but carbon is still present.

The blue trace in Figure 2 shows that almost all impurity peaks were removed after treating the TiO_2_(110) crystals with the wet chemical cleaning procedure described in the experimental details section. This is also supported by the N 1s and C 1s spectra in Figure 3 (blue). After wet-chemically cleaning the crystal, the remaining amount of nitrogen is only 0.17 atomic %, which is less than 5% of the amount of nitrogen in a densely-packed porphyrin layer evaporated in UHV. The remaining number of carbon impurities on the other hand is still around 20 atomic %, more than half the carbon in a densely-packed porphyrin layer (38%). This is unavoidable when working with TiO_2_ under ambient conditions: the surface will always be fully covered with a layer of carboxylic acid impurities, which are present in both water and air, and immediately adsorb onto the reactive surface to cover it completely. The Diebold group studied the impurity-uptake of TiO_2_(110) when exposed to air and water and they found that mainly carboxylic acids, which are present in very small quantities in air, adsorb onto the surface very rapidly [31]. This is in line with the C 1s XPS high-quality measurements of the wet-chemically cleaned crystal in Figure 3. The blue curve, which was recorded after the cleaning procedure, shows peaks at 284.8 and 288.5 eV suggesting that the impurities consist of carboxylic acids as reported by the Diebold group [31,32].

This has direct implications for the adsorption of porphyrins: in contrast to ultrahigh vacuum studies, the porphyrins do not adsorb onto a clean surface, but instead have to replace the impurity molecules that are already adsorbed onto the crystal. For that to happen the porphyrin molecules must bind at least as strong as the impurities to the TiO_2_(110) surface. As discussed above, the impurity species are most likely carboxylic acids, which form covalent bonds to the surface. Porphyrins that only physisorb onto TiO_2_(110), such as 2H-tetraphenylporphyrin, will not be able to replace the carboxylic acid impurities. Instead, porphyrins with linker groups are needed to displace the impurities by forming stronger surface bonds. Porphyrins with phosphonic acid groups are good candidates for a strong bonding molecule [33,34]. However, here we want to compare porphyrins prepared wet chemically with vacuum-deposited porphyrins, and we were not able to evaporate phosphonic acid-functionalized porphyrins without decomposition. Instead, we use the carboxylic-acid-functionalized MCTPP molecule. As the linker group of MCTPP is the same as for the carboxylic acid impurities, we assume that their bond strengths are similar, but because the porphyrin concentration in solution is much higher than that of the impurities, they should be able to replace the carboxylic acid impurities when deposited from solution.

**Figure 4:**
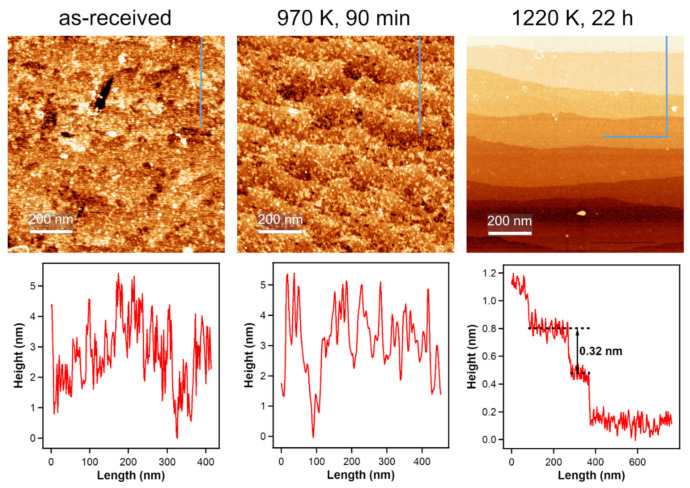
Ambient-condition AFM images of an untreated TiO_2_(110) surface and after annealing in air and corresponding height profiles (indicated by the blue lines in the AFM images). Annealing to 970 K, which is the annealing temperature for UHV-prepared crystals, does not yield flat surfaces. Increasing the annealing temperature to 1220 K for 22 h results in large terraces, indicating atomic ordering.

For all our measurements, we used polished TiO_2_(110) single crystals, but the polished side is—on a microscopic level—still very rough. The left part of Figure 4 shows an ambient condition atomic force microscopy (AFM) image of such a polished surface. In UHV studies, the annealing steps after sputtering the single crystals create very flat surfaces with large terraces [35] and to achieve similar results outside of UHV, we annealed our crystals in a flow of air. Applying the same annealing temperature that we used in our UHV experiments, which is 970 K, for 90 min, does not yield flat surfaces as seen in the middle part of Figure 4. When increasing the annealing temperature to 1220 K for 22 h, large terraces with a step height of 0.32 nm, which is in agreement with literature [36], are formed. This temperature is also employed by other groups to prepare flat surfaces on rutile TiO_2_(110) crystals [37,38,39].

### 2.2. Porphyrin Deposition

**Figure 5:**
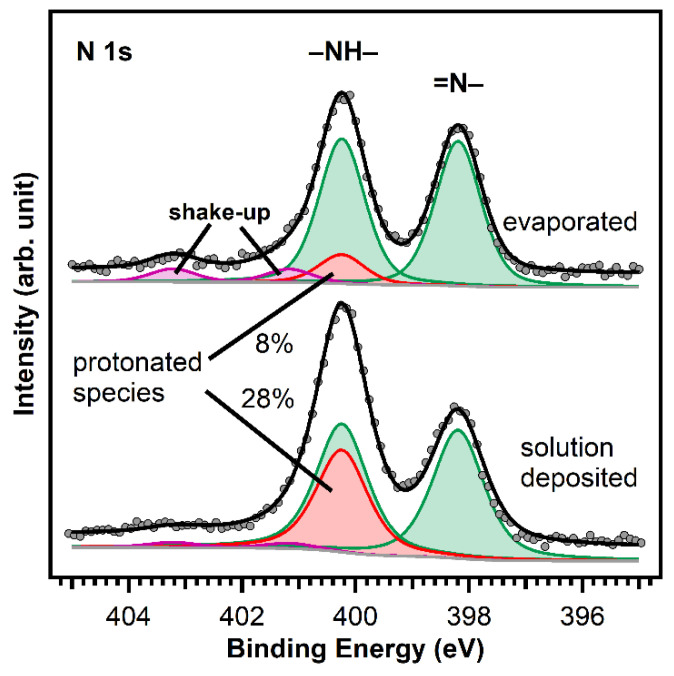
Comparison of the N 1s spectra of MCTPP evaporated (top) and wet-chemically deposited (bottom) onto TiO_2_(110). The two different nitrogen atoms of the porphyrin lead to the two equal intense green peaks: the iminic nitrogen (=N–) atoms to the peak at 398.2 eV and the aminic nitrogen (–NH–) atoms to the peak at 400.2 eV. The red peak is caused by a doubly-protonated porphyrin diacid, which we believe is formed upon interaction of the iminic nitrogen atoms with hydroxyl groups on the surface. The spectra were scaled to the respective porphyrin coverages of 1.0 ML for the evaporated and 1.3 ML for the wet-chemically deposited layers.

As discussed above, we deposited MCTPP from ethanolic solutions. Therefore, after cleaning, the crystals were placed in a 0.01 mM porphyrin solution. Figure 5 shows N 1s spectra of a wet-chemically cleaned crystal that was in contact with a 0.01 mM MCTPP solution for 30 min (bottom), and of a crystal cleaned in ultrahigh vacuum followed by the UHV deposition of MCTPP molecules (top). Both spectra show the same peaks at the same binding energies. It has to be noted that the survey spectrum corresponding to the crystal with the solution-deposited porphyrin molecules does not exhibit any impurity peaks besides small quantities of carbon (see discussion below). The biggest difference between the two measurements is the ratio between the two main nitrogen peaks. Measurements in the past have shown that impurities can adsorb on TiO_2_(110) when brought into contact with solutions [28]. These impurities would appear around 401.2 eV (see yellow peak in Figure 7 below); thus, the increase in the 400.2 eV peak in the solution de-posited spectrum is not due to the presence of nitrogen-containing impurities. We think the reason for the difference is in the different preparation methods employed.

To understand the observed behavior, we have to assign the measured peaks: the green-colored peaks are caused by the two different types of nitrogen atoms that are present in a free-base porphyrin: iminic nitrogen (=N–) leading to the peak at 398.2 eV and aminic nitrogen (–NH–) at 400.2 eV. The barely visible violet peaks are shake-up satellites. The remaining red peak at the same position as the aminic nitrogen peak is typical for porphyrins adsorbed on TiO_2_(110) [23,28,40]. This was first observed in UHV measurements by Lovat et al. [23] and we believe that it is caused by an interaction of the iminic nitrogen atoms with hydroxyl groups on the surface yielding the formation of a doubly-protonated porphyrin diacid. We think that the different ratio between the N 1s peaks of evaporated and solution deposited MCTPP is caused by a higher degree of protonated porphyrins on the wet-chemically prepared crystal. Given that the crystal has been in contact with protic solvents, its surface is probably strongly hydroxylated, and this could lead to a higher degree of protonation. These first results show that it is possible to deposit porphyrins from ethanolic solutions resulting in molecular layers of a similar quality as those obtained by UHV-evaporation but exhibiting a larger degree of protonation.

**Figure 6:**
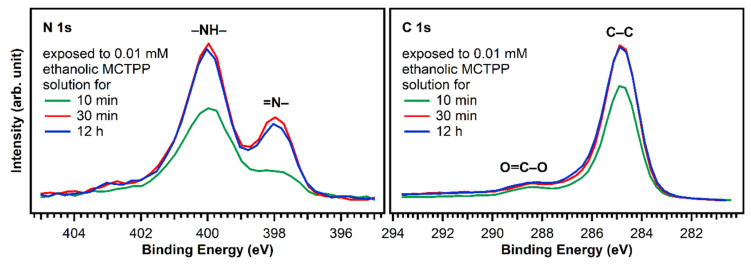
N 1s and C 1s spectra of TiO_2_(110) crystals exposed to ethanolic 0.01 mM MCTPP solution for 10 and 30 min and 12 h. After 30 min, the spectra do not change anymore, suggesting that saturation coverage is reached. The 10 min preparation has less nitrogen and carbon and therefore also a lower porphyrin coverage.

Figure 6 shows N 1s and C 1s spectra of TiO_2_(110) crystals exposed to MCTPP solutions as a function of time. After 30 min, the regions are not changing anymore indicating that a saturation coverage is reached. The saturation coverage at this point is 30% larger (1.3 ML) than a monolayer prepared by MCTPP multilayer-desorption in UHV. We do not expect multilayers to form, because all molecules not directly bound to the surface should be removed by rinsing with the pure solvent. It appears reasonable to assume that the solution-deposited process allows molecules to attach and detach, and thereby reach a more well-ordered and densely-packed structure. In addition, the monolayer reference in UHV was obtained by annealing to above 550 K to desorb multilayers. It is possible that annealing the solution-deposited structure to the same temperature would reduce the coverage slightly, producing the same coverage. It has to be noted, that the coverages were calculated by comparing the N 1s to Ti 2p ratios to those of the monolayer obtained by multilayer desorption. Any impurity on the surface contributing to the C 1s signal will therefore not affect the calculated coverage.

MCTPP evaporated in UHV shows a coverage-dependent change in adsorption geometry. Small submonolayer coverages adsorb with the macrocycle close to the surface, while coverages around 1 ML adopt a more upright-standing adsorption geometry, allowing more molecules to adsorb onto the crystal [28]. We suspect that because of the even higher coverage, the MCTPP molecules adsorbed in the wet-chemically prepared layer are tilted even further away from the surface (close to perpendicular), which would explain the higher coverage.

Finally, it cannot be completely excluded that the evaporation of the pure solvent leaves a small carbon-containing evaporation residue behind. This is consistent with the higher carbon-to-nitrogen ratio observed for the wet-chemically prepared layers compared the layers prepared in UHV. The amount of additional carbon on the wet-chemically prepared layers is roughly 15% of the carbon coverage of a UHV-prepared monolayer.

Considering the full preparation was done outside of ultrahigh vacuum and the crystal was initially fully covered with carboxylic acid impurities, we consider this is a good outcome. In addition, this result is very reproducible allowing us to prepare crystals with adsorbed porphyrins that yield very similar C 1s and N 1s photoelectron spectra. The time the crystal is exposed to solution is important: The carbon-to-nitrogen ratio after 10 min exposure is higher than after 30 min (or longer), indicating that initially more carbon impurities are present. To keep the number of impurities as low as possible, we performed all subsequent experiments on crystals exposed to porphyrin solutions for at least 30 min.

### 2.3. Porphyrin Metalation

After demonstrating that porphyrin layers can be successfully and reliably deposited on TiO_2_(110) following a complete wet chemical route, we will now discuss the metalation behavior of these layers in comparison with UHV prepared porphyrin layers. The metalation reaction is one of the most studied reactions of porphyrins. It has been investigated (1) for dissolved porphyrins in solution [7,41,42,43]; (2) for adsorbed porphyrins in ultrahigh vacuum [20,44,45,46]; and (3) for adsorbed porphyrins exposed to metal ion-containing solutions [28,30,47]. We have therefore decided to use this as our first test reaction.

**Figure 7:**
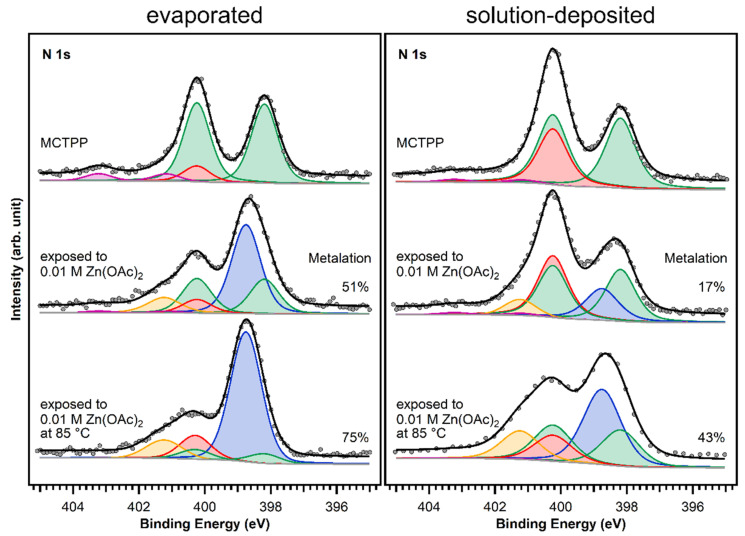
N 1s spectra of evaporated and solution-deposited layers of MCTPP onto TiO_2_(110) before and after metalation at room temperature and at 85 °C with aqueous zinc acetate solution. The blue peak is caused by the metalated porphyrin species and the yellow peak represents impurities that adsorbed during exposure to zinc acetate solution. The spectra were scaled to the respective porphyrin coverages.

The MCTPP-covered crystals were put in aqueous zinc acetate solution with a concentration of 0.01 M for one hour. The results are summarized in Figure 7: the left side shows the measurements performed on sputter-cleaned TiO_2_(110) followed by the UHV evaporation of MCTPP molecules, and the right side shows the spectra of the wet-chemically prepared crystal. The measurements at the top are the ones before metalation, which were already discussed in Figure 6.

After exposing the crystals to zinc acetate solution, the spectral shape changes only slightly in the case of the solution-deposited crystal but very drastically for the vacuum-prepared crystal. Both spectra show an increase at the high binding energy side (orange colored peak). This is the typical binding energy position (401.2 eV) of nitrogen-containing impurities when TiO_2_(110) crystals [28] (and also Au(111) crystals [30]) are exposed to zinc acetate solution. As the solvent was the same that is used during the crystal cleaning, the source of those impurities is most likely the zinc acetate salt. In all cases, the number of impurities from metalating with solution was below 10%.

Larger differences are found at 398.8 eV: fitting the two spectra confirms that the degree of metalation is different. The blue peak, which represents the metalated porphyrin species, is considerably smaller for the wet-chemically prepared crystal: only around 17% of the porphyrin molecules are metalated. However, also on the UHV-prepared crystal the degree of metalation is only around 50%, whereas Franke et al. found for 2HTPP evaporated onto Au(111) complete metalation with zinc acetate solution at room temperature [30]. With the aim to increase the amount of metalated porphyrins, freshly prepared crystals with MCTPP were put in zinc acetate solution for one hour while heating to 85 °C. This leads to a higher degree of metalation on both crystals, but full metalation is still not achieved. On the wet-chemically prepared crystal, the total amount of metalation is only 43% and, therefore, still smaller than on the vacuum-prepared crystal at room temperature where 75% metalation is observed. In all cases, the amount of zinc on the surface is high enough to metalate all present porphyrin molecules and is therefore not the reason for the low degrees of metalation. Both UHV-prepared crystals, the one exposed to zinc acetate solution at room temperature, and the one exposed at 85 °C, have a zinc-to-nitrogen ratio of 0.75. This is three times higher than the value of 0.25, which would be expected for exactly one zinc atom present per porphyrin molecule. The wet-chemically prepared crystal, which was exposed to metal ions at room temperature, shows the same ratio as the UHV-prepared crystals, but the crystal that was metalated at 85 °C has a zinc-to-nitrogen ratio of 2. We see no reason why this sample should contain much more zinc than the other three therefore believe that it is an outlier, probably caused by insufficient rinsing with pure solvent after the metalation reaction.

**Figure 8:**
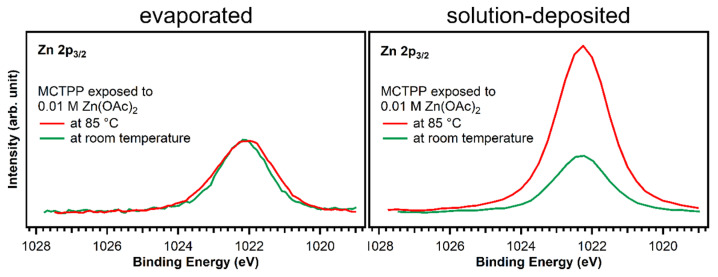
Zn 2p_3/2_ spectra of evaporated and solution-deposited layers of MCTPP onto TiO_2_(110) after metalation at room temperature (green trace) and at 85 °C (red) with aqueous zinc acetate solution. The spectra were scaled to reflect the calculated zinc to nitrogen ratios.

The observed difference in metalation could be related to the packing density present in the saturated monolayers. A porphyrin layer with densely packed upright standing molecules would probably present a lower degree of metalation because the metal ions have to diffuse through the non-polar adsorbate layer to the porphyrin center. We observed a similar effect when we studied the metalation behavior of UHV-prepared MCTPP on TiO_2_(110) as a function of coverage [28,48]. As discussed above, the wet-chemically prepared crystals have a larger surface coverage (1.3 ML) than the UHV prepared crystals (1 ML) and therefore exhibit a lower degree of metalation as observed in Figure 8. This shows that there is a considerable difference between the two preparations. Although the wet chemical route employed to deposit porphyrin layers on TiO_2_(110) produces spectroscopically similar layers to those obtained in UHV, their reactivities towards metalation from solution are different. As discussed above, this might be the result of differences in the arrangement of surface molecules.

## 3. Experimental Details

### 3.1. Wet-Chemically Prepared Crystals

X-ray photoelectron spectroscopy (XPS) measurements were conducted in a Quantera II machine (Physical Electronics, Inc., Chanhassen, MN, USA) with a base pressure below 1 × 10^−9^ mbar. The machine is equipped with a monochromatic Al Kα X-ray source and a dual-beam charge neutralization setup, allowing us to measure non-conductive TiO_2_(110) crystals. The charge neutralization setup slightly overcompensates the charge and shifts all spectra to lower binding energies by around 3 eV. To correct for this, all spectra were shifted to align the main C 1s peak to 284.8 eV. Further information about dual-beam charge neutralization in XPS can be found elsewhere [49].

The cleanliness of glassware and solutions is crucial when working with wet chemical deposition on low-surface-area substrates such as single crystals, where ppb levels of impurities can be enough to completely cover the surface [29,30]. All glass parts that get in contact with either the TiO_2_(110) crystals or the reagent solutions were, therefore, first cleaned with ultrapure water, and then immersed in an aqueous solution of 0.005 M KMnO_4_/0.05 M H_2_SO_4_ for one hour to oxidize organic contaminants. Afterwards, the glass parts were again washed with ultrapure water and moved to a 10% H_2_O_2_ solution for another hour, to remove the remaining KMnO_4_. Finally, after removing the glass parts from the peroxide solution, they were vigorously washed with ultrapure water.

The procedure we used to clean the TiO_2_(110) single crystals is based on a recipe described by the Diebold group [50] and is basically a variation of the “Standard Clean 1” (SC-1) cleaning procedure used to clean wafers [51]: the first step is to sonicate the crystals in 3% soap solution (Labwash premium extra) for 15 min to remove all grease and bigger particles. After sonicating for another 15 min in clean ultrapure water, the crystals are placed in a 65 °C 3:1 mixture of 25% NH_3_ and 30% H_2_O_2_ for 8 min. Finally, the crystals are rinsed and sonicated in ultrapure water again for 15 min. The results of the wet chemical cleaning procedure are discussed in detail in the next section. After cleaning, the crystals are either stored in ultrapure water or directly put into a porphyrin solution.

### 3.2. Ultrahigh-Vacuum-Prepared Crystals

The TiO_2_(110) crystals were cleaned using an established procedure by several cycles of sputtering and annealing [23,28,31], which results in an atomically clean and well-defined surface. The cleanliness was verified by XPS, using a hemispherical SPECS electron analyzer and a monochromatic Al Kα X-ray source in a UHV chamber with a base pressure below 5 × 10^−10^ mbar. To achieve sufficient conductivity for XPS the TiO_2_(110) crystals used for the ultrahigh-vacuum preparations were first annealed in vacuum until they have a light blue color. MCTPP was evaporated onto the clean TiO_2_(110) single crystals from a Knudsen cell. Exposure of the ultrahigh-vacuum-prepared crystals to aqueous zinc acetate solutions was carried out in an argon-filled Teflon liquid cell attached to the UHV chamber. In contrast to the wet-chemically prepared crystals, this crystal was at no point exposed to air during the cleaning process, preparation of the porphyrin layer, transfer to the liquid cell, or exposure to solution. A detailed description of the preparation of the crystals in ultrahigh vacuum can be found elsewhere [28].

### 3.3. Chemicals

The 10 × 10 × 1 mm^3^ rutile TiO_2_(110) crystals were bought from Crystal GmbH (Berlin, Germany). NH_3_ was purchased from VWR Chemicals (Radnor, PE, USA), H_2_O_2_ from BASF (Ludwigshafen, Germany), and ethanol from Fisher Chemicals (Hampton, NH, USA). MCTPP was bought from Porphyrin Laboratories (Scharbeutz, Germany) and Zn(OAc)_2_ from Acros Organics (Phil Lang, NJ, USA). Aqueous zinc acetate solutions were prepared with ultrapure water (MilliQ Synergy UV, 18.2 MΩ cm at 25 °C, <5 ppb TOC).

## 4. Conclusions

We presented a method to clean rutile TiO_2_(110) crystals and deposit molecules from solution, without the need of ultrahigh vacuum. The porphyrin layers are reproducible, of high purity and are composed of mainly free-base porphyrins with some protonated molecules. The amount of protonated porphyrin species obtained in the wet-chemically prepared layer is about three times higher than that present in porphyrin layers deposited in UHV, probably because exposing the crystal to protic solvents results in a stronger hydroxylated surface. Furthermore, we exposed solution-deposited porphyrins to aqueous zinc acetate solutions, which induced partial metalation. In comparison to evaporated porphyrin molecules onto sputter-cleaned TiO_2_(110) crystals, the degree of metalation is considerably smaller, and even after exposure to a zinc acetate solution heated to 85 °C, not even half of the molecules were metalated. This might be due to the different arrangement of molecules on the surface, where molecules deposited from solution form more densely packed layers, which are probably less reactive towards metalation.

## Figures and Tables

**Table 1:** Elemental composition of TiO_2_(110) surfaces as-received, after wet-chemically cleaned and with MCTPP adsorbed from solution and with MCTPP evaporated in UHV. All compositions are given as atomic % within the probed depth.

	TiO_2_(110)		MCTPP + TiO_2_(110)	
	Before Cleaning	Wet-Chemically Cleaned	1.3 ML MCTPP	1.0 ML MCTPP
			Wet-Chemically Prepared	UHV Prepared
Ti	16.04%	24.72%	14.16%	17.05%
O	55.88%	55.21%	36.90%	39.25%
C	23.85%	19.66%	44.64%	37.89%
N	1.20%	0.17%	3.98%	4.06%
Zn	0.12%	<0.03%	<0.05%	<0.02%
Cu	0.82%	<0.02%	<0.04%	<0.02%
Ca	0.58%	<0.01%	<0.20%	<0.40%
S	0.33%	<0.05%	<0.01%	<0.20%
P	0.57%	<0.06%	<0.01%	<0.30%
Si	0.61%	<0.08%	<0.01%	<0.80%

## Data Availability

The data presented in this study are available on request from the corresponding author.

## Sample Availability

Samples of the compounds are not available from the authors.

## References

[1] Fleischer E.B. (1970). Structure of Porphyrins and Metalloporphyrins. Acc. Chem. Res..

[2] Milgrom L.R. (1997). The Colours of Life: An Introduction to the Chemistry of Porphyrins and Related Compounds.

[3] Battersby A.R. (2000). Tetrapyrroles: The pigments of life. Nat. Prod. Rep..

[4] Kadish K., Smith K.M., Guilard R. (1999). Biochemistry and Binding: Activation of Small Molecules.

[5] Kadish K., Smith K.M., Guilard R. (2002). Chlorophylls and Bilins: Biosynthesis, Synthesis and Degradation.

[6] Hodgkin D.C., Kamper J., Mackay M., Pickworth J., Trueblood K.N., White J.G. (1956). Structure of vitamin B_12_. Nature.

[7] Guilard R., Kadish K.M. (1988). Some aspects of organometallic chemistry in metalloporphyrin chemistry: Synthesis, chemical reactivity, and electrochemical behavior of porphyrins with metal-carbon bonds. Chem. Rev..

[8] Smith P.D., James B.R., Dolphin D.H. (1981). Structural aspects and coordination chemistry of metal porphyrin complexes with emphasis on axial ligand binding to carbon donors and mono- and diatomic nitrogen and oxygen donors. Coord. Chem. Rev..

[9] Vicente M.D., Smith K.M. (2014). Syntheses and Functionalizations of Porphyrin Macrocycles. Curr. Org. Synth..

[10] Mochida I., Suetsugu K., Fujitsu H., Takeshita K., Tsuji K., Sagara Y., Ohyoshi A.A. (1982). Kinetic-Study on Reduction of Nitric-Oxide over Cobalt Tetraphenylporphyrin Supported on Titanium-Dioxide. J. Catal..

[11] Paddock R.L., Hiyama Y., McKay J.M., Nguyen S.T. (2004). Co(III) porphyrin/DMAP: An efficient catalyst system for the synthesis of cyclic carbonates from CO_2_ and epoxides. Tetrahedron Lett..

[12] Berg K., Selbo P.K., Weyergang A., Dietze A., Prasmickaite L., Bonsted A., Engesaeter B.O., Angell-Petersen E., Warloe T., Frandsen N. (2005). Porphyrin-related photosensitizers for cancer imaging and therapeutic applications. J. Microsc..

[13] Sharman W.M., Allen C.M., van Lier J.E. (1999). Photodynamic therapeutics: Basic principles and clinical applications. Drug Discov. Today.

[14] Filippini D., Alimelli A., Di Natale C., Paolesse R., D’Amico A., Lundstrom I. (2006). Chemical sensing with familiar devices. Angew. Chem. Int. Ed..

[15] Rakow N.A., Suslick K.S. (2000). A colorimetric sensor array for odour visualization. Nature.

[16] Vilmercati P., Cudia C.C., Larciprete R., Cepek C., Zampieri G., Sangaletti L., Pagliara S., Verdini A., Cossaro A., Floreano L. (2006). Molecular Orientations, Electronic Properties and Charge Transfer Timescale in a Zn-Porphyrin/C_70_ Donor–Acceptor Complex for Solar Cells. Surf. Sci..

[17] Campbell W.M., Burrell A.K., Officer D.L., Jolley K.W. (2004). Porphyrins as light harvesters in the dye-sensitised TiO_2_ solar cell. Coord. Chem. Rev..

[18] Auwärter W., Seufert K., Klappenberger F., Reichert J., Weber-Bargioni A., Verdini A., Cvetko D., Dell’Angela M., Floreano L., Cossaro A. (2010). Site-Specific Electronic and Geometric Interface Structure of Co-Tetraphenyl-Porphyrin Layers on Ag(111). Phys. Rev. B.

[19] Beggan J.P., Krasnikov S.A., Sergeeva N.N., Senge M.O., Cafolla A.A. (2012). Control of the axial coordination of a surface-confined manganese (III) porphyrin complex. Nanotechnology.

[20] Gottfried J.M., Flechtner K., Kretschmann A., Lukasczyk T., Steinrück H.-P. (2006). Direct synthesis of a metalloporphyrin complex on a surface. J. Am. Chem. Soc..

[21] Bürker C., Franco-Cañellas A., Broch K., Lee T.L., Gerlach A., Schreiber F. (2014). Self-Metalation of 2H-Tetraphenylporphyrin on Cu(111) Studied with XSW: Influence of the Central Metal Atom on the Adsorption Distance. J. Phys. Chem. C.

[22] Röckert M., Franke M., Tariq Q., Ditze S., Stark M., Uffinger P., Wechsler D., Singh U., Xiao J., Marbach H. (2014). Coverage- and temperature-dependent metalation and dehydrogenation of tetraphenylporphyrin on Cu(111). Chem. Eur. J..

[23] Lovat G., Forrer D., Abadia M., Dominguez M., Casarin M., Rogero C., Vittadini A., Floreano L. (2015). Hydrogen capture by porphyrins at the TiO_2_(110) surface. Phys. Chem. Chem. Phys..

[24] Werner K., Mohr S., Schwarz M., Xu T., Amende M., Döpper T., Görling A., Libuda J. (2016). Functionalized Porphyrins on an Atomically Defined Oxide Surface: Anchoring and Coverage-Dependent Reorientation of MCTPP on Co_3_O_4_(111). J. Phys. Chem. Lett..

[25] Olszowski P., Zajac L., Godlewski S., Such B., Pawlak R., Hinaut A., Jöhr R., Glatzel T., Meyer E., Szymonski M. (2017). Ordering of Zn-centered Porphyrin and Phthalocyanine on TiO2(011): STM Studies. Beilstein J. Nanotechnol..

[26] Afzal S., Daoud W.A., Langford S.J. (2013). Photostable self-cleaning cotton by a copper(II) porphyrin/TiO_2_ visible-light photocatalytic system. ACS Appl. Mater. Interfaces.

[27] Tu W., Dong Y., Lei J., Ju H. (2010). Low-potential photoelectrochemical biosensing using porphyrin-functionalized TiO_2_ nanoparticles. Anal. Chem..

[28] Wechsler D., Fernández C.C., Steinrück H.-P., Lytken O., Williams F.J. (2018). Covalent Anchoring and Interfacial Reactions of Adsorbed Porphyrins on Rutile TiO_2_(110). J. Phys. Chem. C.

[29] Franke M., Marchini F., Jux N., Steinrück H.-P., Lytken O., Williams F.J. (2016). Zinc Porphyrin Metal-Center Exchange at the Solid-Liquid Interface. Chem. Eur. J..

[30] Franke M., Marchini F., Steinrück H.-P., Lytken O., Williams F.J. (2015). Surface Porphyrins Metalate with Zn Ions from Solution. J. Phys. Chem. Lett..

[31] Balajka J., Hines M.A., DeBenedetti W.J.I., Komora M., Pavelec J., Schmid M., Diebold U. (2018). High-affinity adsorption leads to molecularly ordered interfaces on TiO_2_ in air and solution. Science.

[32] Schnadt J., O’Shea J.N., Patthey L., Schiessling J., Krempaský J., Shi M., Mårtensson N., Brühwiler P.A. (2003). Structural study of adsorption of isonicotinic acid and related molecules on rutile TiO_2_(110) II: XPS. Surf. Sci..

[33] Galoppini E. (2004). Linkers for anchoring sensitizers to semiconductor nanoparticles. Coord. Chem. Rev..

[34] Brennan B.J., Llansola Portoles M.J., Liddell P.A., Moore T.A., Moore A.L., Gust D. (2013). Comparison of silatrane, phosphonic acid, and carboxylic acid functional groups for attachment of porphyrin sensitizers to TiO_2_ in photoelectrochemical cells. Phys. Chem. Chem. Phys..

[35] Dulub O., Valentin C.D., Selloni A., Diebold U. (2006). Structure, defects, and impurities at the rutile TiO_2_(011)-(2 × 1) surface: A scanning tunneling microscopy study. Surf. Sci..

[36] Diebold U. (2003). The Surface Science of Titanium Dioxide. Surf. Sci. Rep..

[37] Sanz M., Walczak M., Oujja M., Cuesta A., Castillejo M. (2009). Nanosecond pulsed laser deposition of TiO_2_: Nanostructure and morphology of deposits and plasma diagnosis. Thin Solid Films.

[38] Mostéfa-Sba H., Domenichini B., Bourgeois S. (1999). Iron deposition on TiO_2_(110): Effect of the surface stoichiometry and roughness. Surf. Sci..

[39] Xue S., Sasahara A., Onishi H. (2020). Atomic-scale topography of rutile TiO_2_(110) in aqueous solutions: A study involving frequency-modulation atomic force microscopy. J. Chem. Phys..

[40] Köbl J., Wang T., Wang C., Drost M., Tu F., Xu Q., Ju H., Wechsler D., Franke M., Pan H. (2016). Hungry Porphyrins: Protonation and Self-Metalation of Tetraphenylporphyrin on TiO2(110)-1 × 1. ChemistrySelect.

[41] Lavallee D.K. (1986). Complexation and Demetalation Reactions of Porphyrins. Comments Inorg. Chem..

[42] Lavallee D.K., White A., Diaz A., Battioni J.-P., Mansuy D. (1986). Efficient metalloporphyrin synthesis under mild conditions using N-benzylporphyrins. Tetrahedron Lett..

[43] Singh R., Geetanjali K. (2005). Novel synthetic methodology for metalloporphyrins in ionic liquid. J. Braz. Chem. Soc..

[44] Diller K., Klappenberger F., Marschall M., Hermann K., Nefedov A., Woll C., Barth J.V. (2012). Self-metalation of 2H-tetraphenylporphyrin on Cu(111): An x-ray spectroscopy study. J. Chem. Phys..

[45] Wechsler D., Franke M., Tariq Q., Zhang L., Lee T.-L., Thakur P.K., Tsud N., Bercha S., Prince K.C., Steinrück H.-P. (2017). Adsorption Structure of Cobalt Tetraphenylporphyrin on Ag(100). J. Phys. Chem. C.

[46] Gottfried J.M. (2015). Surface chemistry of porphyrins and phthalocyanines. Surf. Sci. Rep..

[47] Fernández C.C., Spedalieri C., Murgida D.H., Williams F.J. (2017). Surface Influence on the Metalation of Porphyrins at the Solid–Liquid Interface. J. Phys. Chem. C.

[48] Wechsler D., Vensaus P., Tsud N., Steinrück H.-P., Lytken O., Williams F.J. (2021). Surface Reactions and Electronic Structure of Carboxylic Acid Porphyrins Adsorbed on TiO_2_(110). J. Phys. Chem. C.

[49] Larson P.E., Kelly M.A. (1998). Surface charge neutralization of insulating samples in x-ray photoemission spectroscopy. J. Vac. Sci. Technol. A.

[50] Müllner M., Balajka J., Schmid M., Diebold U., Mertens S.F.L. (2017). Self-Limiting Adsorption of WO_3_ Oligomers on Oxide Substrates in Solution. J. Phys. Chem. C.

[51] Kern W., Puotinen D.A. (1970). Cleaning solutions based on hydrogen peroxide for use in silicon semiconductor technology. RCA Rev..

